# Urinary F2-Isoprostanes and Metabolic Markers of Fat Oxidation

**DOI:** 10.1155/2015/729191

**Published:** 2015-02-23

**Authors:** Dora Il'yasova, Lynne E. Wagenknecht, Ivan Spasojevic, Steven Watkins, Donald Bowden, Frances Wang, Ralph B. D'Agostino

**Affiliations:** ^1^School of Public Health, Georgia State University, 140 Decatur Street, Urban Life Building, Atlanta, GA 30303, USA; ^2^Wake Forest School of Medicine, Public Health Sciences, Winston-Salem, NC 27157, USA; ^3^Duke Cancer Institute, Duke University Medical Center, 2424 Erwin Road, Durham, NC 27705, USA; ^4^Lipomics Technologies, Division of Metabolon, 3410 Industrial Boulevard, West Sacramento, CA 95691, USA; ^5^Center for Genomics and Personalized Medicine Research, Wake Forest School of Medicine, Winston-Salem, NC 27157, USA

## Abstract

Metabolomic studies of increased fat oxidation showed increase in circulating acylcarnitines C2, C8, C10, and C12 and decrease in C3, C4, and C5. We hypothesize that urinary F2-isoprostanes reflect intensity of fatty acid oxidation and are associated with circulating C2, C8, C10, and C12 directly and with C3, C4, and C5 inversely. Four urinary F2-isoprostane isomers and serum acylcarnitines are quantified using LC-MS/MS within the Insulin Resistance Atherosclerosis Study nondiabetic cohort (*n* = 682). Cross-sectional associations between fasting urinary F2-isoprostanes (summarized as a composite index) and the selected acylcarnitines are examined using generalized linear models. F2-isoprostane index is associated with C2 and C12 directly and with C5 inversely: the adjusted beta coefficients are 0.109, 0.072, and −0.094, respectively (*P* < 0.05). For these acylcarnitines and for F2-isoprostanes, the adjusted odds ratios (ORs) of incident diabetes are calculated from logistic regression models: the ORs (95% CI) are 0.77 (0.60–0.97), 0.79 (0.62–1.01), 1.18 (0.92–1.53), and 0.51 (0.35–0.76) for C2, C12, C5, and F2-isoprostanes, respectively. The direction of the associations between urinary F2-isoprostanes and three acylcarnitines (C2, C5, and C12) supports our hypothesis. The inverse associations of C2 and C12 and with incident diabetes are consistent with the suggested protective role of efficient fat oxidation.

## 1. Introduction

Urinary F2-isoprostanes are validated indices of human oxidative status, reflecting an overall generation of free radicals, among them reactive oxygen species (ROS) [1–3]. Commonly, any statistically significant elevation of systemic F2-isoprostanes (by any amount) is interpreted as harmful oxidative stress [1]. A prominent example is the conventional view that the elevated F2-isoprostane levels in obesity represent obesity-induced oxidative stress and a mechanistic link between obesity and cardiovascular risk [4]. In contrast, our research found that elevated urinary F2-isoprostanes levels present a favorable trait predicting lower risks of weight gain and type 2 diabetes [5, 6]; and the findings on the risk of weight gain have been independently confirmed in another cohort [7]. To reconcile the two aspects of urinary F2-isoprostanes, that is, as favorable metabolic trait and as systemic indices of ROS, we hypothesized that mitochondrial oxidative metabolism is the main determinant of urinary F2-isoprostane levels [8], because mitochondria are the major endogenous source of ROS [9]. We also hypothesized that fatty acid oxidation plays the predominant role in the connection between mitochondrial metabolism and urinary F2-isoprostane levels, because mitochondrial fatty acid oxidation (a) produces higher levels of ROS [10] and (b) largely accounts for the peripheral tissue oxidative metabolism [11]. This hypothesis builds upon the protective roles that the intensive mitochondrial metabolism, fatty acid oxidation, and physical activity play against obesity and type 2 diabetes [12] and the well-known fact that physical activity induces increases in F2-isoprostane levels [13, 14].

In earlier publications, we examined several epidemiological evidences supporting the hypothesized connection between urinary F2-isoprostanes and fat oxidation. We found that a racial group with lower levels of fat oxidation, that is, African Americans [15], congruently has lower levels of urinary F2-isoprostanes [16]. We also demonstrated that fasting levels of nonesterified fatty acids, which are known to stimulate muscle fat oxidation, correlate directly with urinary F2-isoprostanes [17]. Further exploration of our hypothesis has become possible due to the recent metabolomic studies of circulating metabolites in conditions with intensified fat oxidation, namely, during fasting and moderate intensity exercise [18, 19]. Specifically, the metabolomic study of fasting demonstrated that circulating levels of C3 (propionylcarnitine), C4 (butyrylcarnitine), and C5 (valerylcarnitine), all derivatives of amino acid oxidation, decline, whereas C2 (acetylcarnitine) levels increase [18]. The study of moderate intensity exercise found that a 60–120 min run resulted in a transient increase of C8 (octanoylcarnitine), C10 (decanoylcarnitine), and C12 (dodecanoylcarnitine) levels [19]. If urinary F2-isoprostanes correlate with the intensity of fat oxidation, it is logical to expect that these biomarkers are associated with C2, C8, C10, and C12 directly and with C3, C4, and C5 inversely. We also hypothesized that these same acylcarnitines are associated with incident type 2 diabetes congruently with the inverse association between F2-isoprostanes and type 2 diabetes [6]. We explored these questions by examining both F2-isoprostane and acylcarnitine data from the Insulin Resistance Atherosclerosis Study (IRAS) multiethnic cohort.

## 2. Research Design and Methods

### 2.1. Study Population

The IRAS is a well-characterized multiethnic cohort described in detail [17]. The analytical cohort includes the baseline nondiabetic participants with normal (NGT) or impaired glucose tolerance (IGT) only, with available measurements of urinary F2-isoprostanes and acylcarnitines (*n* = 682), among them 114 participants developing type 2 diabetes during the follow-up.

### 2.2. Measurements

All subjects fasted for 12 hours and refrained from heavy exercise, smoking, and alcohol consumption for 24 hours before the visit. The metabolic and anthropometric measurements were described earlier [17]. Participants had a frequently sampled glucose tolerance test for which insulin sensitivity (*S*_I_) and acute insulin response (AIR) were calculated. Four isomers of F2-isoprostanes—iPF2*α*-III, 2,3-dinor-iPF2*α*-III, iPF2*α*-VI, and 8,12-iso-iPF2*α*-VI—are quantified in morning spot urine samples (stored at −70°C) by liquid chromatography with tandem mass spectrometry detection (LC-MS/MS) as previously described. F2-isoprostane levels are corrected by urinary creatinine to account for differences in urine dilution [3].

Acylcarnitines are also measured by LC-MS/MS. Deuterium-labeled internal standards were added to 25 microliters of serum and the mixture was solubilized in methanol followed by a crash extraction and then injected onto an Atlantis HILIC Column connected to a Waters Xevo triple quadrupole mass spectrometer (Waters, MA). Acylcarnitines were ionized via positive electrospray and the mass spectrometer was operated in the tandem MS mode. The absolute concentration of each acylcarnitine is determined by comparing the corresponding peak to that of the relevant internal standard.

### 2.3. Statistical Analysis

Spearman correlation coefficient is used to examine crude correlations between acylcarnitines. For F2-isoprostanes, we calculated a composite index that ranks individuals based on all four measurements: [(X1_*i*_ − M1)/SD1 + (X2_*i*_ − M2)/SD2 + (X3_*i*_ − M3)/SD3 + (X4_*i*_ − M4)/SD4]/4, where “*i*” is a notation for a participant; values of four F2-isoprostanes species (X1–4) were standardised by subtracting estimated mean (M1–4) and divided by standard deviation (SD1–4). For acylcarnitines, we use natural-log transformed variables to reduce the influence of high values.

Adjusted beta coefficients for the associations between F2-isoprostane index and the acylcarnitines are calculated from generalized linear models. The minimally adjusted models include demographic variables (age, gender, and race/ethnicity) and BMI; the fully adjusted models include additional metabolic variables (IGT, insulin sensitivity [log⁡(*S*_I_ + 1)], and AIR). The association between the acylcarnitines and incident diabetes (*n* = 114) is quantified by odds ratios calculated from logistic regression models, with the covariates selected by our previously published analysis [6].

## 3. Results

Our study population is metabolically and ethnically diverse, with 43% Non-Hispanic and 32% Hispanic Whites and 25% African Americans and 26% normal, 44% overweight, and 29% obese (Table 1). One third of the study population is NGT with two-thirds being IGT. The observed correlations between the examined acylcarnitines are in agreement with the findings from the metabolomics studies [18, 19]. As expected, the strongest correlations are found between C8, C10, and C12 (0.82 ≤ *r* ≤ 0.97, *P* < 0.05) and between C3, C4, and C5 (0.43 ≤ *r* ≤ 0.59, *P* < 0.05). C2 correlates most strongly with C12 (*r* = 0.41, *P* < 0.05) as compared to other acylcarnitines.

Examining the associations between fasting levels of F2-isoprostanes (expressed as composite index) and acylcarnitines, we find that beta coefficients are consistently positive for the associations with C2, C8, C10, and C12, whereas for C3, C4, and C5 the beta coefficients are negative (Table 2). Three acylcarnitines show statistically significant associations: *P* values for the beta coefficients are 0.03 for C2, C5, and C12. The magnitude of the beta coefficients and the standard errors are generally consistent between the minimally and the fully adjusted models (Table 2). For example, beta coefficients for the association between F2-isoprostanes and C2 correspond to 9.8% and 10.9% increase of mean F2-isoprostane index per standard deviation of C2 distribution.

The three acylcarnitines that are significantly associated with F2-isoprostanes (C2, C5, and C12) are examined as predictors of incident diabetes (Table 3). C2 and C12 are inversely associated with type 2 diabetes risk (*P* values 0.03 and 0.06, resp.); and C3 shows a nonsignificant positive association (*P* value 0.19). The estimated magnitudes of the associations with C2 and C12 are very close (odds ratios are 0.77 and 0.79, resp.). As expected, the association with the composite index of F2-isoprostanes confirms the inverse relationships with type 2 diabetes risk in this analytical cohort (Table 3).

## 4. Discussion

This analysis is inspired by the recent metabolomic studies that examined changes in circulating metabolites within the physiological conditions incurring increased fat oxidation [18, 19]. We hypothesized that urinary F2-isoprostane levels reflect a greater ability to oxidize fat and therefore are associated directly with C2, C8, C10, and C12 and inversely with C3, C4, and C5. The detected trends are consistent with our hypotheses. Significant associations are found for urinary F2-isoprostanes C2, C12, and C5. Acylcarnitine C5 is a metabolite related to catabolism of branched chain amino acids (BCAAs), which is thought to be negatively regulated by mitochondrial fatty acid oxidation [20]. In fact, the metabolomic study of fasting showed an increase in circulating levels of BCAAs, which could result from a decreased catabolism of BCAAs in the condition of increased fat oxidation [18]. Acylcarnitine C2, while potentially derived from multiple fuel sources that generate acetyl-CoA, is known as a metabolite increased by exercise and fasting due in large part to increased fatty acid oxidation [21, 22]. Importantly, the detected associations between F2-isoprostane and acylcarnitines are stable, as shown by the minimally and fully adjusted models in Table 2. This in conjunction with no findings contradicting our hypothesis insures the consistency of these results.

Additional support for our hypothesis comes from the prospective analysis of type 2 diabetes risk. Our results indicate a trend of inverse associations of C2 and C12 with incident type 2 diabetes, whereas C5 shows an opposite trend. These findings are in agreement with the expectations based on the results in Table 2, demonstrating the overall consistency of our results. They also support the suggested protective role of efficient fat oxidation in etiology of type 2 diabetes. Importantly, this analysis utilizes the data from the well-characterized multiethnic cohort, which provides an opportunity to adjust the results for potential confounders and assumes generalizability. In summary, our results provide additional support to the hypothesis that urinary levels of F2-isoprostanes are related to the intensity of fatty acid oxidation.

## Figures and Tables

**Table 1:** Characteristics of the study population (*n* = 682).

Characteristics	Mean (SD) or number (%)
Age (years)	54.5 (8.4)
Sex, female (number, %)	385 (56.5)
Ethnicity (number, %)	
Non-Hispanic White	291 (43)
African American	174 (25)
Hispanic White	217 (32)
Normal glucose tolerance, NGT (number, %)	226 (33)
Impaired glucose tolerance IGT (number, %)	456 (67)
BMI (kg/m^2^)	
Normal (<25)	179 (26)
Overweight (25–29.9)	303 (44)
Obese (≥30)	199 (29)
Missing	1
iPF2*α*-III (ng/mg creatinine)	0.25 (0.20)
2,3-dinor-iPF2*α*-III (ng/mg creatinine)	4.32 (3.05)
iPF2*α*-VI (ng/mg creatinine)	6.46 (4.08)
8,12-iso-iPF2*α*-VI (ng/mg creatinine)	4.19 (2.92)
F2-isoprostane composite index	1.41 (0.80)
Acetylcarnitine, C2 (nmole/g)	7.16 (2.45)
Propionylcarnitine, C3 (nmole/g)	0.37 (0.12)
Butyrylcarnitine, C4 (nmole/g)	0.19 (0.11)
Valerylcarnitine, C5 (nmole/g)	0.15 (0.05)
Octanoylcarnitine, C8 (nmole/g)	0.17 (0.12)
Decanoylcarnitine, C10 (nmole/g)	0.33 (0.29)
Dodecanoylcarnitine, C12 (nmole/g)	0.21 (0.11)

Mean (SD) presented for continuous variables; F2-isoprostane composite index was calculated using all four F2-isoprostane measurements as follows: each value was standardized (divided by the standard deviation) and mean of the four standardized values was calculated for each participant.

**Table 2:** Association between F2-isoprostane index and selected acylcarnitines (*n* = 682).

Acylcarnitines	Beta coefficient (95% CI)	
	Minimally adjusted model	Fully adjusted model
Acetylcarnitine, C2	**0.098 (0.042, 0.154)**	**0.109 (0.051, 0.167)**
Propionylcarnitine, C3	−0.027 (−0.089, 0.034)	−0.024 (−0.088, 0.040)
Butyrylcarnitine, C4	−0.010 (−0.067, 0.048)	−0.010 (−0.069, 0.049)
Valerylcarnitine, C5	**−0.093 (−0.156, −0.030)**	**−0.094 (−0.159, −0.029)**
Octanoylcarnitine, C8	0.010 (−0.046, 0.066)	0.014 (−0.044, 0.073)
Decanoylcarnitine, C10	0.016 (−0.040, 0.071)	0.020 (−0.039, 0.079)
Dodecanoylcarnitine, C12	**0.065 (0.009, 0.122)**	**0.072 (0.013, 0.132)**

Beta coefficients show differences in F2-isoprostanes index associated with a change in acylcarnitines (log-transformed) equal to standard deviation. Minimally adjusted models included the demographic variables (age, gender, and race/ethnicity) and BMI; fully adjusted models included additional metabolic variables (IGT-status, insulin sensitivity, and AIR). Statistically significant results (*P* < 0.05) are in bold.

**Table 3:** Association of the baseline F2-isoprostane index and three selected acylcarnitines with incident type 2 diabetes (114 cases).

	OR (95% CI)
F2-isoprostane composite index	0.51 (0.35, 0.76)
Acetylcarnitine, C2	0.77 (0.60, 0.97)
Valerylcarnitine, C5	1.18 (0.92, 1.53)
Dodecanoylcarnitine, C12	0.79 (0.62, 1.01)

ORs adjusted for age, gender, ethnicity, clinic and baseline BMI, and IGT-status and scaled to SD of F2-isoprostanes or acylcarnitines (log-transformed).

## Abbreviations

AIR: Acute insulin response
BMI: Body mass index
C2: Acetylcarnitine
C3: Propionylcarnitine
C4: Butyrylcarnitine
C5: Valerylcarnitine
C8: Octanoylcarnitine
C10: Decanoylcarnitine
C12: Dodecanoylcarnitine
CI: Confidence interval
IGT: Impaired glucose tolerance
IRAS: Insulin Resistance Atherosclerosis Study
NGT: Normal glucose tolerance
LC-MS/MS: Liquid chromatography with tandem mass spectrometry
OR: Odds ratio
*S*_I_: Insulin sensitivity.
